# Editorial: Environmental bioremediation: application of enzymes and microbes

**DOI:** 10.3389/fbioe.2023.1327124

**Published:** 2023-11-15

**Authors:** Xiyu Cheng, Abdur Rahim Khan, Karima ELKarrach, Feng Wang

**Affiliations:** ^1^ College of Life Sciences and Bioengineering, School of Physical Science and Engineering, Beijing Jiaotong University, Beijing, China; ^2^ Department of Plant Pathology, College of Agricultural and Environmental Sciences, University of California, Davis, Davis, CA, United States; ^3^ Ecole des Hautes Etudes de Biotechnologie et de santé, Casablanca, Morocco; ^4^ School of Food and Biological Engineering, Jiangsu University, Zhenjiang, China

**Keywords:** bioremediation, soil, water, enzymes, microbes

Soil and water contamination due to organic or inorganic pollutants has long been a major environmental problem and is attracting increasing attention (Hu et al., 2023; Thacharodi et al., 2023). Different pollutants have been reported to be detrimental to public health. For example, crude oil contamination occurs during the processes of petroleum exploration, transportation, storage, and refining. Similarly, many other chemicals (i.e., polychlorinated biphenyl (PCBs), polycyclic aromatic hydrocarbons (PAHs), pesticides, *etc.*), synthetic plastics, and pharmaceuticals in wastewaters are not completely removed by conventional treatment plants (Gosai et al., 2022; Thacharodi et al., 2023). Therefore, it is necessary to develop an efficient strategy to remediate water and soil contaminated with these diverse pollutants.

Bioremediation of these refractory pollutants using microorganisms such as bacteria, fungi, yeasts, and algae has recently obtained enormous interest worldwide due to its efficiency, low cost, and sustainability (Gosai et al., 2022; Tomar et al., 2022; Aminian-Dehkordi et al., 2023). Technological innovation and mechanism exploration are of great importance to improve the environmental bioremediation efficiency and to reduce the corresponding health risks. Ma et al. developed a synergistic removal technique for refractory pentachlorobenzene (PeCB) using *Pseudomonas* sp. JS100 coupled with immobilized nanoscale zero-valent iron (NZVI). The observation of structural and textural features verified that zero-valent iron particles were dispersed and attached to the biofilter, hence improving the specific surface area to 34.5 m^2^ g^−1^. The removal efficiency of PeCB reached as high as 80.2% within 48 h. In the system, PeCB was decomposed by NZVI into lower chlorobenzenes, and then utilized by *Pseudomonas* sp. JS100 as nutrients. As a result, rapid and efficient removal of PeCB was observed.

Chitosan, a biopolymer material obtained from marine biomass wastes, has great potential for wastewater treatment and soil remediation because of its good biocompatibility and degradability. Zhang et al. reported a natural chitin degrading bacterium (*Bacillus cereus* ZWT-08) from coastal mud that operates by re-screening the chitin deacetylase activity. ZWT-08 was cultured in an optimized fermentation medium with 1% (m/V) glucose and yeast extract at pH 6.0, 37°C, and a stirring speed of 180 r/min, and the deacetylation activity of the supernatant reached 613.25 U/mL. The removal rate of the acetyl groups in this chitin substrate digested by chitin deacetylase from ZWT-08 reached 89.29%. The obtained chitosan produced in this process has a degree of deacetylation higher than 90%. The higher the degree of deacetylation, the better the adsorption performance of chitosan. This work provided a promising avenue for the production of valuable chitosan and its future application in waste treatment.

Bioinformatics technology has greatly deepened our understanding of bioremediation processes. Champramary et al. investigated the mycoremediation potential of armillarioids by using a comparative genomics analysis. Genes involved in mycoremediation were identified to confine the distinctive bioremediation capabilities of the armillarioids. Their studies underlined the distinct, increased potential of aromatic-degrading genes/enzymes in armillarioids, with a particular emphasis on a high copy number and a diverse spectrum of benzoate 4-monooxygenase [EC:1.14.14.92] homologs. Other enzymes involved in the degradation of different monocyclic aromatics were more abundant in the armillarioids than in other white-rot basidiomycetes. Several genes that were involved in the degradation of benzoates and other monocyclic aromatics were found to be remarkably expressed in wood-invading fungal mycelia by performing a transcriptome analysis of *A. ostoyae* and *A. borealis* isolates. These results provide a useful tool for screening promising fungal candidates in bioremediation based on their genomics data.

Metformin, which is globally used to treat type II diabetes, is a major anthropogenic pollutant to be bioremediated. Martinez-Vaz et al. isolated two metformin-biodegrading bacteria from a wastewater treatment plant. The authors found that metformin was stoichiometrically metabolized to guanylurea by *Aminobacter* sp. MET, while *Pseudomonas mendocina* MET completely degraded metformin. Genome analysis indicated that genes involved in the transport of guanylurea in *Aminobacter* sp. MET were expressed heterologously and were shown to serve as an antiporter to expel the toxic guanidinium compound. In addition, the authors obtained a novel guanylurea hydrolase enzyme in *P. mendocina* MET.

The studies in this Research Topic report the latest developments in some bioremediation technologies that enable efficient treatment of various pollutants. However, bioremediation of refractory pollutants such as PCBs, PAHs, pesticides, synthetic plastics, and pharmaceuticals remains a tough challenge. Transgenic, hetero microbial, sequential anaerobic and aerobic, immobilized enzymes, nano-remedial approaches, and their combinations are a few examples of recent, cutting-edge techniques to treat many organic and inorganic pollutants (Hu et al., 2023). In the future, it is critical to comprehend the microbial diversity of polluted sites and to investigate the biodegradable potential of culture-independent microorganisms as well as the fungal-bacterial interactions in these degradation processes by using advanced techniques (i.e., metagenomics) (Gosai et al., 2022; Win and Song, 2023). Given the rapid development of systems and synthetic biology (Aminian-Dehkordi et al., 2023), the design of tailor-made organisms for bioremediation is expected to be enabled, as well as the creation of cost-effective artificial microbial cell factories in natural settings for efficient removal of these refractory pollutants. Extensive research should be focused on the above aspects to harness the full potential of bioremediation technology.
